# Risk of Osteoporosis and Anemia in Plant-Based Diets: A Systematic Review of Nutritional Deficiencies and Clinical Implications

**DOI:** 10.7759/cureus.88461

**Published:** 2025-07-21

**Authors:** Folasade E Akinwumi, Amos O Akinyemi, Benjamin Akangbe, Oluwasanmi M Odeniran, Johnathan Sehkar, Christiana O Olajimbiti, Oluwatobi H Ajayi

**Affiliations:** 1 National Centre for Food Manufacturing, University of Lincoln, Lincoln, GBR; 2 Toxicology and Cancer Biology, University of Kentucky, Lexington, USA; 3 Public Health, Georgia State University, Atlanta, USA; 4 Nutrition, Dietetics, and Sensory Sciences, Kansas State University, Manhattan, USA; 5 Health and Clinical Sciences, University of Kentucky, Kentucky, USA; 6 Veterinary Pathology, Univeristy of Abuja, Abuja, NGA; 7 Duke Human Vaccine Institute, Duke University School of Medicine, Durham, USA

**Keywords:** anemia, deficiency, osteoporosis, plant-based diet, vegans, vegetarians

## Abstract

The global shift toward plant-based diets is accelerating, driven by growing awareness of health, environmental, and ethical concerns. While these diets are linked to reduced risks of chronic diseases, emerging evidence highlights potential nutritional deficiencies, particularly in calcium, iron, and vitamin B12, that may compromise bone and hematologic health. This systematic review investigates the relationship between strict plant-based dietary practices and the risks of anemia and osteoporosis. Following PRISMA guidelines, we conducted a comprehensive search across four databases PubMed, Scopus, Web of Science, and Google Scholar using relevant search terms including "plant-based diet", "vegan", "vegetarian", "anemia", "osteoporosis", "vitamin B12 deficiency", "iron deficiency", and "calcium deficiency". We retrieved 1290 records; after removing 210 duplicates and screening 1080 records, 208 full-text articles were assessed. Ultimately, 76 studies met the eligibility criteria and were included in the review. Our synthesis reveals consistent evidence linking poorly planned plant-based diets to increased risk of iron-deficiency anemia and reduced bone mineral density. These findings show the importance of nutritional education, regular monitoring, and appropriate supplementation to support individuals following vegan or vegetarian diets in achieving long-term health.

## Introduction and background

Introduction

In recent years, plant-based diets have witnessed a remarkable surge in popularity, particularly across Western countries. Between 2014 and 2017, the number of vegans in the United States rose dramatically from approximately four million to 19.7 million, representing a 500% increase [1, 2]. Similar trends are evident in the United Kingdom, Germany, and Italy, where a growing portion of the population has adopted vegetarian and vegan lifestyles [2]. This global dietary shift is driven by a combination of factors, including health consciousness, environmental sustainability, ethical concerns related to animal welfare, and religious or cultural practices [3].

Plant-based diets are defined as dietary patterns that emphasize foods derived primarily from plants, such as vegetables, fruits, legumes, nuts, seeds, oils, and whole grains, and they are associated with several health benefits [4, 5]. Epidemiological studies suggest that plant-based diets may reduce the risk of chronic conditions, including cardiovascular disease, obesity, type 2 diabetes, and certain cancers [6, 7]. Additionally, the ecological footprint of plant-based food production is substantially lower than that of animal agriculture, making these diets appealing to environmentally conscious consumers [8].

Within the plant-based dietary spectrum, vegan diets represent the most restrictive form, as they exclude all animal-derived products, including meat, poultry, fish, dairy, eggs, and even animal by-products such as gelatin or honey. In contrast, vegetarian diets are more inclusive, typically allowing for dairy (lacto-vegetarian), eggs (ovo-vegetarian), or both (lacto-ovo vegetarian). Some vegetarians may also occasionally include fish (pescatarian) or even small amounts of meat on rare occasions, though they still identify with predominantly plant-based eating. The degree of restriction varies based on ethical, cultural, religious, or health-related motivations, with veganism often linked to animal welfare and environmental concerns, while vegetarianism may be more flexible and focused on personal health or tradition [7, 9]. To replicate the sensory and nutritional qualities of meat and dairy, a diverse range of plant-based alternatives has been developed. Common meat substitutes are made from soy, pea protein, wheat gluten (seitan), or mushrooms, each chosen for their texture, protein content, or culinary versatility [10, 11]. Dairy alternatives, such as almond, oat, soy, or rice milk, are increasingly consumed by individuals with lactose intolerance, dairy allergies, or ethical concerns [10]. Despite these innovations and the increasing accessibility of plant-based foods, there are growing concerns about potential nutritional inadequacies, especially when diets are followed without adequate planning [10, 11]. Nutrients of concern include iron, calcium, and vitamin B12-micronutrients that play essential roles in hematologic function and bone health but are either absent or less bioavailable in many plant-based sources [12, 13].

Iron deficiency is particularly prevalent among vegans due to the lower bioavailability of non-heme iron found in plant foods compared to heme iron from animal sources. This can increase the risk of iron-deficiency anemia, a condition marked by fatigue, impaired oxygen transport, and diminished cognitive and physical performance [14-16]. Similarly, vitamin B12, which is almost exclusively found in animal products, is essential for red blood cell production and neurological health; its deficiency is common in long-term vegans and can lead to megaloblastic anemia and neuropathy [17-19]. Calcium intake may also be insufficient, particularly in the absence of fortified foods or supplements, elevating the risk of osteoporosis, a skeletal disorder characterized by reduced bone mass and increased fracture susceptibility [20-22].

This systematic review aims to explore the relationship between strict plant-based dietary practices and the risk of anemia and osteoporosis. It draws upon 76 peer-reviewed studies identified through a structured search strategy and applies the PRISMA framework to assess study quality and relevance. We highlight the nutritional vulnerabilities associated with plant-based eating patterns and discuss their clinical implications. This review seeks to inform healthcare professionals and diet-conscious individuals about the importance of dietary planning, monitoring, and supplementation to minimize long-term health risks while reaping the benefits of plant-based nutrition.

Methods overview

**Figure 1:**
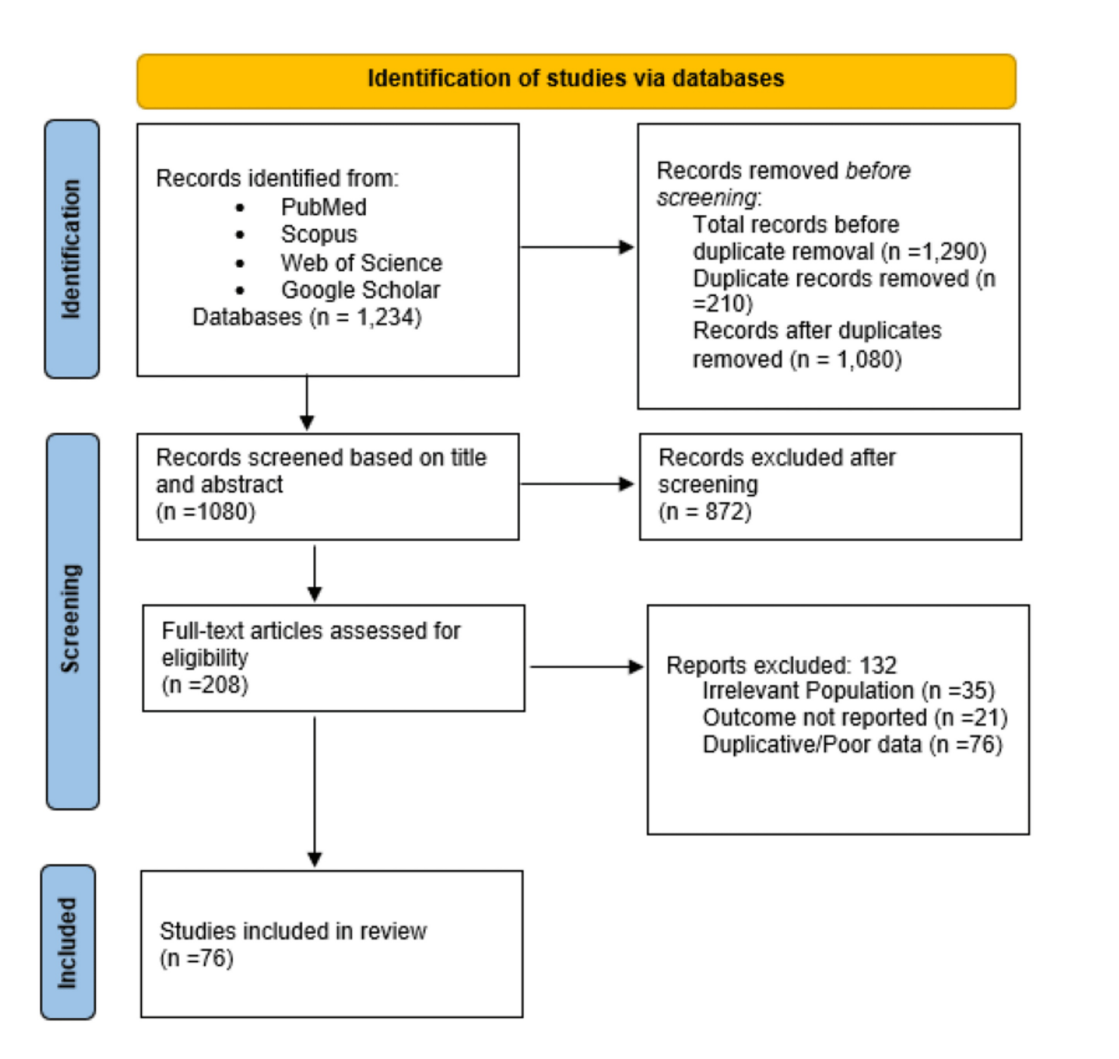
Flow diagram depicting the study selection process for the systematic review on plant-based diets and the risk of anemia and osteoporosis

This systematic review aims to critically assess the association between strict plant-based diets and the risk of anemia and osteoporosis, with particular focus on nutritional gaps in calcium, vitamin B12, and iron intake. Following the Preferred Reporting Items for Systematic reviews and Meta-Analyses (PRISMA) guidelines, we performed a structured literature search across four major databases: PubMed, Scopus, Web of Science, and Google Scholar, covering studies published from January 2000 to February 2025. We used the following combination of search terms: "plant-based diet" OR "vegan" OR "vegetarian" AND "anemia" OR "iron deficiency" OR "vitamin B12 deficiency" OR "osteoporosis" OR "calcium deficiency". We identified 1290 records, removed 210 duplicates, screened 1080 titles/abstracts, assessed 208 full-text articles, and excluded 132 articles (for reasons including irrelevant population (n=35), outcome not reported (n=21), and duplicative or poor data (n=76)). The final number of studies included in the review is 76. The full PRISMA flowchart is included as Figure 1, and a comprehensive list of all 76 studies included in the final analysis is provided in Table 1, with proper citation and summary of findings.

**Table 1:** : List of articles included in the systematic review on the risk of osteoporosis and anemia in plant-based diets

	Article title	Reference
1	Foods for plant-based diets: challenges and innovations	Alcorta et al., 2021 [1]
2	The Vegan Society and social movement professionalization, 1944–2017	Wrenn, 2019 [23]
3	Characteristics of Americans choosing vegetarian and vegan diets for health reasons	Cramer et al., 2017 [3]
4	Healthy plant-based diet: what does it really mean?	Williams et al., 2017 [4]
5	Vegetarian diets, low-meat diets and health: a review	McEvoy et al., 2012 [24]
6	Physiological and dietary determinants of iron status in Spanish vegetarians	Gallego-Narbón et al., 2019 [25]
7	Public awareness of a plant-based diet following the release of "Game Changers" and "What The Health" documentaries	Hartwell et al., 2022 [26]
8	Iron status of vegans, vegetarians and pescatarians in Norway	Henjum et al., 2021 [14]
9	Plant-based dairy and the food transformation: leveraging sociocultural acceptability and personal benefit to shift consumer behavior toward sustainability	Järvinen, 2024 [27]
10	A plant-based dietary intervention improves beta-cell function and insulin resistance in overweight adults: a 16-week randomized clinical trial	Kahleova et al., 2018 [5]
11	Zinc: an essential but elusive nutrient	King, 2011 [28]
12	A global analysis of national dietary guidelines on plant-based diets and substitutions for animal-based foods	Klapp et al., 2022 [29]
13	Sustaining protein nutrition through plant-based foods	Langya et al., 2021 [30]
14	Vegetarian and plant-based diets in health and disease prevention	Moretti, 2017 [31]
15	Zinc pharmacotherapy for elderly osteoporotic patients with zinc deficiency in a clinical setting	Nakano et al., 2021 [32]
16	Isolation and analysis of vitamin B12 from plant samples	Nakos et al., 2017 [33]
17	Nutrient intake and status in adults consuming plant-based diets compared to meat-eaters: a systematic review	Neufingerl et al., 2021 [34]
18	Vegetarian dietary patterns for adults: a position paper of the Academy of Nutrition and Dietetics	Raj, 2025 [22]
19	Gen Z's willingness to adopt plant-based diets: empirical evidence from Greece, India, and the UK	Raptou et al., 2024 [35]
20	Meeting the nutrient reference values on a vegetarian diet	Reid et al., 2013 [36]
21	Vitamin B12 among vegetarians: status, assessment and supplementation	Rizzo et al., 2016 [37]
22	Osteoporosis prevention, diagnosis, and therapy	NIH Consensus Development Panel on Osteoporosis Prevention, Diagnosis, and Therapy, 2001 [38]
23	Healthful and unhealthful plant-based diets and the risk of coronary heart disease in US adults	Satija et al., 2017 [6]
24	Vegetarian, vegan diets and multiple health outcomes: a systematic review with meta-analysis of observational studies	Dinu et al., 2017 [7]
25	Food in the Anthropocene: the EAT–Lancet Commission on healthy diets from sustainable food systems	Willet et al., 2019 [39]
26	Protein adequacy, plant protein proportion, and main plant protein sources consumed across vegan, vegetarian, pescovegetarian, and Semivegetarian diets: a systematic review	Roland et al., 2025 [9]
27	Rethinking food and agriculture 2020-2030: the second domestication of plants and animals, the disruption of the cow, and the collapse of industrial livestock farming	Tubb et al., 2021 [40]
28	Fermentation of plant-based milk alternatives for improved flavour and nutritional value	Tangyu et al., 2019 [41]
29	Fatty acid profile and cardiometabolic markers in relation with diet type and omega-3 supplementation in Spanish vegetarians	Salvador et al., 2019 [42]
30	Plant-based meat alternatives: technological, nutritional, environmental, market, and social challenges and opportunities	Andreani et al., 2023 [11]
31	Position of the academy of nutrition and dietetics: vegetarian diets	Melina et al., 2016 [12]
32	Impact of vegan and vegetarian diets on neurological health: a critical review	Clemente-Suárez, 2025 [15]
33	Vegetarian diet - how does it affect our body?	Tekiela, 2025 [16]
34	Vitamin B12 status in vegan and vegetarian Seventh-day Adventists: a systematic review and meta-analysis of serum levels and dietary intake	Janko et al., 2025 [17]
35	Vitamin B12: as important to pediatricians as geriatricians. 2025.	Borowitz, 2025 [18]
36	Exploring vitamin B12 supplementation in the vegan population: a scoping review of the evidence	Fernandes et al., 2024 [19]
37	The effects of vegetarian diets on bone health: a literature review	Falchetti et al., 2022 [20]
38	UK clinical guideline for the prevention and treatment of osteoporosis	Gregson et al., 2022 [21]
39	Red and processed meats and health risks: how strong is the evidence?	Qian et al., 2020 [43]
40	Applied animal ethics in industrial food animal production: exploring the role of the veterinarian	Hernandez et al., 2022 [44]
41	Comparison of nutritional quality of the vegan, vegetarian, semi-vegetarian, pesco-vegetarian and omnivorous diet	Clarys et al., 2014 [45]
42	Health effects of vegan diets	Craig, 2009 [46]
43	Amino acids: metabolism, functions, and nutrition	Wu, 2009 [47]
44	Dietary protein quality evaluation in human nutrition. Report of an FAQ Expert Consultation	FAO, 2013 [48]
45	Assessment of protein adequacy in developing countries: quality matters	Ghosh et al., 2012 [49]
46	Nutrient density and nutritional value of meat products and non-meat foods high in protein	Bohrer, 2017 [50]
47	Eating to live well—or worse? The role of vegan and vegetarian diets in mental health	Dobersek et al., 2025 [51]
48	Protein intake among community-dwelling older adults: the influence of (pre-) motivational determinants	Verwijs et al., 2022 [52]
49	Dietary protein: an essential nutrient for bone health	Bonjour, 2005 [53]
50	Vitamin B12 in health and disease	O'Leary et al., 2010 [54]
51	Vitamin B12 and folate status in Spanish lacto-ovo vegetarians and vegans	Gallego-Narbón et al., 2019 [55]
52	Vitamin B12-containing plant food sources for vegetarians	Watanabe et al., 2014 [56]
53	Vitamin B12 in foods, food supplements, and medicines—a review of its role and properties with a focus on its stability	Temova Rakuša et al., 2022 [57]
54	Vitamin D and immune function	Prietl et al., 2013 [58]
55	Vitamin D deficiency	Holick, 2007 [59]
56	Sunlight and vitamin D: a global perspective for health	Wacker et al., 2013 [60]
57	Vitamin D intake: a global perspective of current status	Calvo et al., 2005 [61]
58	Iron bioavailability from food fortification to precision nutrition. A review	Blanco-Rojo et al., 2019 [62]
59	Iron deficiency	Parischa, 2021 [63]
60	Iron bioavailability and dietary reference values	Hurrell et al., 2010 [64]
61	Nutritional iron deficiency	Zimmermann et al., 2007 [65]
62	Vegetarian diets across the lifecycle: impact on zinc intake and status	Foster et al., 2015 [66]
63	Vitamin D intake and status in children and adolescents: Comparing vegetarian, vegan, and omnivorous diets	Devulapalli, 2025 [67]
64	Scientific Opinion on Dietary Reference Values for fats, including saturated fatty acids, polyunsaturated fatty acids, monounsaturated fatty acids, trans fatty acids, and cholesterol	EFSA Panel on Dietetic Products, 2010 [68]
65	Food fortification through innovative technologies	Alina et al., 2019 [69]
66	The epidemiology of osteoporosis	Clynes et al., 2020 [70]
67	Vegetarian diets and bone status	Tucker, 2014 [71]
68	Plant foods rich in antioxidants and human cognition: a systematic review	Baroni et al., 2021 [72]
69	The ethics of veganism	Jaiswal et al., 2024 [73]
70	A scoping review of the environmental impacts and nutrient composition of plant-based milks	Berardy et al., 2022 [74]
71	Plant-based food and protein trend from a business perspective: markets, consumers, and the challenges and opportunities in the future	Aschemann-Witzel et al., 2021 [75]
72	Consumer preference segments for plant-based foods: The role of product category	Cardellio et al., 2022 [76]

## Review

Shifting consumer preferences toward plant-based diets

**Figure 2:**
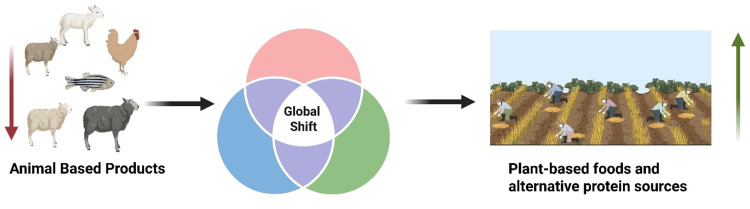
Illustration of consumer shift to plant-based diet Figure created by the authors

Over the past decade, there has been a notable transformation in dietary preferences as more individuals transition to plant-based diets. This shift is driven by a confluence of health motivations, ethical considerations, and environmental concerns [35]. Consumers are increasingly aware of the links between excessive consumption of animal-based foods and chronic diseases such as cardiovascular disease, type 2 diabetes, and certain cancers [43]. As a result, many are embracing plant-based eating patterns in pursuit of better health outcomes [1, 24]. The growing awareness of climate change and the environmental burden of livestock farming, responsible for significant greenhouse gas emissions, land use, and water depletion, has further fueled the adoption of vegetarian and vegan lifestyles [77]. Plant-based diets are considered more sustainable, offering a lower environmental impact compared to omnivorous diets [1]. Moreover, ethical issues surrounding animal welfare and factory farming practices have reinforced the moral rationale for avoiding animal products, particularly among younger generations [44]. This trend has been supported by an increase in the availability and variety of plant-based products. The food industry has responded rapidly, introducing fortified plant milks, plant-based meats, and algae-derived omega-3 supplements to help consumers meet nutritional needs while avoiding animal sources [42]. Additionally, social media, health documentaries, and high-profile endorsements from athletes and celebrities have further normalized plant-based lifestyles and contributed to their global appeal [26] (Figure 2). Despite its growing popularity, the shift to plant-based diets raises concerns about potential nutrient deficiencies, especially in populations that adopt these diets without proper guidance [3]. Nutrients such as vitamin B12, iron, calcium, zinc, and omega-3 fatty acids (docosahexaenoic acid (DHA) and eicosapentaenoic acid (EPA)) are less bioavailable or present in limited quantities in plant foods [24]. Without adequate planning or supplementation, individuals, particularly pregnant women, children, and older adults, may face increased health risks, including anemia, reduced bone density, and impaired neurological function [24, 25]. Therefore, nutrition education and public health strategies must accompany the dietary transition to ensure that plant-based diets are not only ethical and sustainable but also nutritionally adequate.

Nutritional deficiencies

**Figure 3:**
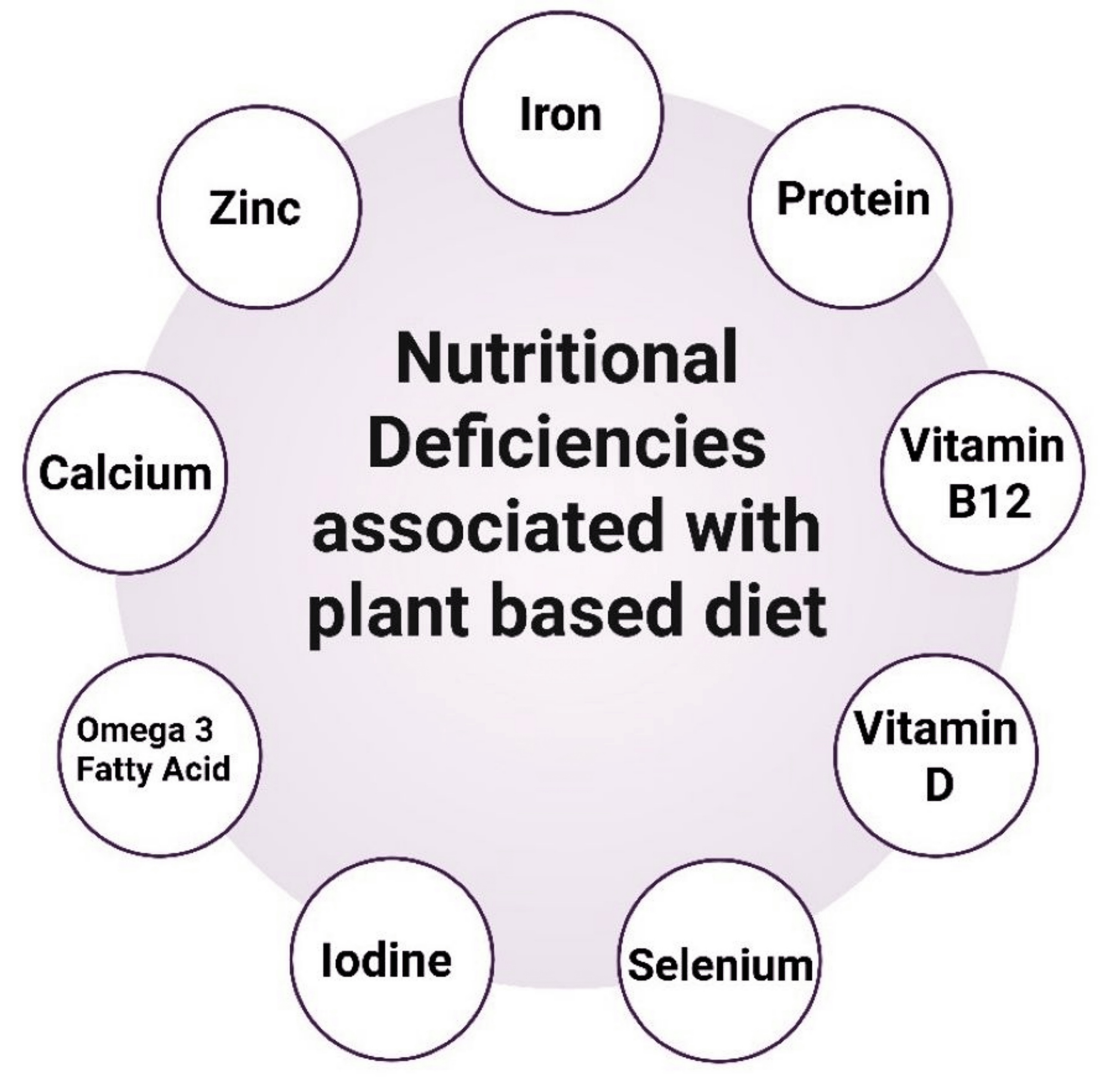
Illustrating the nutritional deficiencies commonly associated with plant-based diets Figure created by the authors

Plant-based diets, though rich in fiber, antioxidants, and phytochemicals, may fall short in providing certain essential nutrients due to the absence or low bioavailability of key components found predominantly in animal-derived foods. Individuals following vegan or vegetarian diets are at a higher risk of nutrient deficiencies, particularly in protein, vitamin B12, iron, calcium, zinc, omega-3 fatty acids, and vitamin D [45, 46]. This is largely attributed to the lower density of some nutrients in plant foods and the presence of compounds like phytates and oxalates that impair absorption [1]. Without careful planning and supplementation, these deficiencies can impact immune function, bone health, muscle maintenance, and neurological performance. Below shows the systematic breakdown of nutrient deficiencies commonly observed in plant-based diets (Figure 3).

Protein

Proteins are fundamental macromolecules required for cellular structure, enzymatic function, hormone production, and immune defense [47]. While the body can synthesize non-essential amino acids, essential amino acids must be obtained from dietary sources. The quality of a protein is determined by its amino acid composition and digestibility [48]. Animal proteins generally provide all essential amino acids in adequate amounts, whereas many plant proteins, especially cereals and legumes, lack sufficient quantities of lysine, threonine, and tryptophan [49, 50]. Vegetarians often consume less protein than omnivores [34], and the lower biological value of plant-based proteins has been a concern. However, evidence shows that a well-planned vegetarian or vegan diet, when diversified to include various plant protein sources, can provide adequate protein and prevent deficiency [12, 30, 36]. Furthermore, processing techniques such as fermentation, sprouting, and cooking improve protein quality by reducing anti-nutrients and enhancing digestibility [30]. Adequate protein intake is critical for maintaining bone health, as it supports bone matrix synthesis and calcium retention [52, 53]. Higher protein consumption is also associated with improved muscle strength and suppression of parathyroid hormone levels, which may otherwise promote bone resorption [78].

Vitamin B12

Vitamin B12 (cobalamin) is an essential water-soluble micronutrient involved in DNA synthesis, red blood cell formation, and neurological function [54]. Deficiency can lead to megaloblastic anemia and irreversible neurological damage, manifesting in cognitive impairments such as memory loss, confusion, and even dementia in severe cases [55]. Cobalamin is synthesized exclusively by microorganisms in the digestive tracts of animals or through industrial fermentation [56]. Consequently, it is naturally present only in animal-derived foods such as meat, eggs, and dairy products [79]. Individuals following plant-based diets are at higher risk of deficiency due to the absence of bioavailable vitamin B12 in unfortified plant foods [33, 37, 56]. Although some fermented and plant-based foods like chlorella, nori, or spirulina have been explored as B12 sources, these either contain inactive analogues or provide unreliable amounts [1, 56]. Fortified foods such as breakfast cereals, plant-based milks, and nutritional yeast are essential for vegetarians to meet daily cobalamin needs [57, 80].

However, up to 50% of vitamin B12 may be lost during food processing and cooking [1], emphasizing the importance of proper food handling and supplementation. Furthermore, ageing is associated with a decline in intrinsic factor production and absorption capacity in the gut, making elderly individuals more susceptible to deficiency even with adequate intake [55]. As a result, experts recommend that older adults consume more than the standard recommended dietary allowance (RDA) or rely on supplements with active B12 (methylcobalamin or cyanocobalamin) [54]. Emerging technologies aim to improve access to B12 in sustainable ways. Hydroponic cultivation systems have been shown to enrich vegetables like soybean sprouts, lettuce, and radish with vitamin B12 when grown in nutrient-supplemented water [1]. Other innovations include using *Propionibacterium* during fermentation to enhance B12 content in foods such as sauerkraut or fenugreek juice [41]. While such strategies are promising, supplementation remains the most reliable means of preventing deficiency in plant-based populations [37]. Due to its water solubility, vitamin B12 has low toxicity potential, as excess amounts are excreted in urine [37]. This makes pharmacological supplementation both safe and essential in preventing deficiency, particularly in vulnerable groups.

Vitamin D

Vitamin D is a critical fat-soluble vitamin essential for calcium absorption and bone mineralization [58]. Beyond its classical role in musculoskeletal health, vitamin D modulates immune responses, influences cell differentiation and proliferation, and is involved in several metabolic pathways [58]. It plays an integral role in preventing disorders such as rickets, osteomalacia, and osteoporosis by promoting calcium and phosphate homeostasis [59]. Although sunlight is a free and abundant source of vitamin D, cutaneous synthesis is influenced by several factors, including age, skin pigmentation, geographic location, clothing habits, and sunscreen use, which may impair its production [60]. As a result, dietary intake becomes crucial to meet physiological requirements. However, few foods naturally contain vitamin D, mainly fatty fish, liver, eggs, and fortified dairy products, putting individuals following plant-based diets at a higher risk of deficiency [81]. Studies have consistently shown lower serum 25(OH)D concentrations in vegans and vegetarians compared to omnivores, making supplementation or fortified food consumption vital for these populations [82]. Plant-based consumers are therefore encouraged to monitor their vitamin D status and consume fortified plant-based milks, cereals, or vitamin D supplements, particularly during the winter months or in regions with limited sunlight. Additionally, biofortification strategies and the use of lichen-derived vitamin D₂ or D₃ supplements offer promising solutions for vegan consumers [61].

Iron

Iron is a trace element necessary for oxygen transport (via hemoglobin), energy metabolism, DNA synthesis, and numerous enzymatic reactions involved in growth, immune defense, and neural development [62]. It is also essential for collagen synthesis and interacts with vitamin B-complex to maintain bone metabolism. Insufficient iron intake leads to symptoms ranging from fatigue and reduced immunity to iron deficiency anemia, impaired physical performance, and poor cognitive outcomes [63]. Iron exists in two forms in the diet: heme iron from animal sources and non-heme iron from plant-based foods. The bioavailability of heme iron is significantly higher and less affected by dietary inhibitors compared to non-heme iron, whose absorption can be impaired by phytates, polyphenols, and calcium [64] (Table 2, Figure 3). Consequently, vegetarians must consume more iron and strategically combine iron-rich foods with enhancers such as vitamin C, citric acid, and certain fermented products to improve absorption [31]. Interestingly, studies suggest that vegetarians often maintain adequate iron levels despite lower dietary intake due to increased non-heme iron absorption under conditions of low iron stores, a homeostatic adaptation of the body [65]. However, subpopulations such as menstruating women, children, and individuals with gastrointestinal disorders remain at higher risk of iron deficiency and should be monitored closely [25].

**Table 2:** Comprehensive overview of the pros and cons of plant-based diets

Aspect	Pros	Cons
Health benefits	Associated with reduced risk of cardiovascular disease, obesity, hypertension, and type 2 diabetes [1].	Higher risk of deficiencies in vitamin B12, calcium, omega-3 fatty acids (DHA/EPA), and iron if not supplemented [24].
Bone health	Higher intake of fruits and vegetables may improve potassium, magnesium, and vitamin K, supporting bone metabolism [25].	Lower intake of calcium, protein, vitamin B12, and zinc can increase risk of osteoporosis [70].
Cognitive development	Rich in antioxidants and anti-inflammatory compounds that may support brain health [72].	Low levels of DHA and EPA may impair cognitive and neurological function, particularly in children and pregnant women [42].
Environmental impact	Reduced greenhouse gas emissions, land and water use; supports biodiversity [1].	Production of ultra-processed plant-based foods can have hidden environmental costs [1].
Ethical considerations	Eliminates harm to animals; aligns with moral and ecological values [73].	Ethical constraints may limit dietary variety or cause social discomfort in mixed-diet settings [27].
Accessibility	Plant-based options and fortified foods are increasingly available in stores and restaurants [74].	Accessibility varies by region; fortified products may be expensive or unavailable in low-income communities [29].
Public perception	Promoted by social media, public health campaigns, and lifestyle influencers [42].	May be perceived as restrictive or elitist; social stigma in certain cultures [84].
Economic impact	Shifting demand has opened new market opportunities in plant-based industries and food innovation [75].	Meat and dairy industries may face economic disruption; job loss in traditional agriculture sectors [40].
Long-term sustainability	Encourages sustainable agriculture and climate-resilient food systems.	Needs continuous innovation to improve nutritional profile and sustainability of substitutes.

Zinc

Zinc is a fundamental micronutrient required for catalytic activity of over 300 enzymes, supporting roles in DNA synthesis, immune function, wound healing, growth, and bone development [83]. Zinc deficiency can present with alopecia, dermatitis, poor wound healing, impaired taste and smell, growth retardation, and immune dysfunction. It also plays an important role in regulating osteoblastic activity and bone mineral density. Zinc bioavailability is lower in plant-based diets due to the presence of phytates in legumes and whole grains that bind zinc and inhibit its absorption [28]. Therefore, vegetarians and vegans are advised to consume zinc-rich foods such as legumes, nuts, seeds, whole grains, tofu, and dairy (for vegetarians), and to consider fortified foods or supplements if necessary. Emerging evidence suggests that zinc supplementation may aid in fracture healing and bone regeneration, particularly in older adults with osteoporosis or zinc deficiency [34]. Clinical studies have shown that zinc can improve bone mineral density and reduce fracture risk, making it a candidate for adjunctive therapy in managing osteoporosis [32]. Thus, adequate zinc intake should be prioritized in populations at risk of deficiency or with elevated bone health needs.

Omega-3 fatty acids

Omega-3 fatty acids, primarily alpha-linolenic acid (ALA), are converted into the long-chain polyunsaturated fatty acids, docosahexaenoic acid (DHA) and eicosapentaenoic acid (EPA), which are crucial for optimal cardiovascular, cognitive, immune, and neurological function [42]. A balanced ratio of omega-6 to omega-3 fatty acids is necessary for maintaining proper eicosanoid production, yet vegetarian diets tend to be disproportionately high in omega-6 and low in omega-3, placing vegetarians at risk of suboptimal DHA and EPA levels (McEvoy et al., 2022). Particularly for children and pregnant women, who have higher physiological demands, supplementation may be necessary to prevent developmental and health complications [24]. The European Food Safety Authority recommends a daily intake of 2-4 grams of EPA and DHA combined for cardiovascular health, and a minimum of 250 mg daily to support normal cardiac function [68]. Although ALA can be sourced from plant-based foods such as flaxseeds, walnuts, leafy greens, and hemp seeds, the body's conversion rate to DHA and EPA is inefficient. Consequently, alternative sources such as microalgae-based supplements are becoming increasingly vital for vegan and vegetarian populations to meet omega-3 requirements [42].

Calcium

Calcium is the most abundant mineral in the human body, with 99% stored in bones and teeth and the remaining 1% circulating in blood and tissues [69]. Calcium plays a fundamental role in bone structure, neuromuscular activity, and intracellular signaling. Globally, calcium deficiency contributes significantly to osteoporosis and fracture risk, affecting millions annually [1]. Vegetarians, particularly vegans, are more prone to calcium deficiency due to limited access to dairy products, which are the primary sources of dietary calcium. Plant-based alternatives such as fortified plant milks are essential, yet still may not fully meet daily requirements unless carefully monitored [55]. Calcium absorption can also be hindered by inhibitors like phytates and oxalates, commonly found in plant foods, while factors such as age, hormonal status, and lifestyle further influence bioavailability [1] (Table 3). Excessive intake of sodium, phosphorus, and caffeine may increase urinary calcium excretion, further exacerbating the deficiency. While higher protein intake has been associated with increased calcium loss, some studies suggest that vegetarians with lower protein consumption may consequently require lower calcium intakes, though this remains debated.

**Table 3:** Comparison of nutrient content and bioavailability in plant-based vs animal-based foods DHA - docosahexaenoic acid; EPA - eicosapentaenoic acid

Nutrient	Animal-based sources	Plant-based sources	Bioavailability
Protein	Meat, poultry, eggs, dairy, fish	Legumes, tofu, soy, nuts, seeds, whole grains	Animal protein is complete; most plant proteins are incomplete (except soy, quinoa).
Vitamin B12	Meat, dairy, eggs, fish	Not naturally present (only in fortified foods/supplements)	Exclusively from animal sources; vegans require supplementation.
Iron	Red meat, liver, poultry, fish	Lentils, spinach, tofu, quinoa, fortified cereals	Heme iron (animal) has higher absorption than non-heme iron (plant).
Zinc	Beef, poultry, shellfish	Beans, whole grains, nuts, seeds	Plant-based zinc is less bioavailable due to phytates that inhibit absorption.
Calcium	Milk, cheese, yogurt	Fortified plant milks, tofu, leafy greens, almonds	Oxalates and phytates in plants reduce absorption.
Omega-3 (EPA/DHA)	Fatty fish (salmon, sardines)	Flaxseed, chia seeds, walnuts, microalgae	Plants offer ALA, but conversion to EPA/DHA is inefficient; microalgae is a direct source.
Vitamin D	Fortified milk, fish liver oils, eggs	Fortified plant milks, UV-exposed mushrooms	D3 (animal) is more effective than D2 (plant); supplementation often needed.
Iodine	Fish, dairy, eggs, iodized salt	Seaweed, iodized salt	Content in seaweed varies; risk of deficiency without fortified sources.
Vitamin A	Liver, dairy, eggs (preformed retinol)	Carrots, sweet potatoes, leafy greens (beta-carotene)	Animal-based vitamin A is readily absorbed; plant beta-carotene requires conversion.
Selenium	Fish, eggs, meat	Brazil nuts, whole grains, sunflower seeds	Plant selenium content depends on soil; Brazil nuts are an excellent source.

Osteoporosis and bone health

Bone is a metabolically active tissue that undergoes constant remodeling, necessitating adequate nutrients for mineralization and strength. Nutrients such as calcium, protein, vitamin D, zinc, magnesium, and vitamin K are all essential for optimal bone health [70]. Osteoporosis, a condition characterized by decreased bone mineral density and increased fracture risk, affects over 200 million people worldwide, especially among postmenopausal women and the elderly [38]. In the United States alone, the economic burden of osteoporosis-related fractures is estimated at $17.9 billion annually [70]. Although the etiology of osteoporosis includes genetic, hormonal, and lifestyle factors, nutritional status remains central. Vegetarian diets, if not well planned, may fall short in key bone-related nutrients, including calcium, vitamin B12, zinc, copper, and protein, thereby contributing to increased osteoporosis risk [71]. However, high intake of fruits and vegetables among vegetarians can offer protective effects through increased intake of potassium, vitamin K, and magnesium. Vitamin K is known to regulate osteocalcin, a protein involved in bone formation, and has been associated with reduced fracture risk. Magnesium supports calcium transport and contributes to bone structural integrity. Thus, while plant-based diets may elevate risk for certain deficiencies, strategic nutritional planning can help preserve bone health.

Clinical implications and nutritional management of micronutrient deficiencies in plant-based diets

Recent clinical evidence emphasizes the growing need to monitor and manage micronutrient deficiencies among individuals who follow plant-based diets. While vegetarian and vegan dietary patterns have been associated with reduced risks of chronic diseases such as cardiovascular disease and certain cancers, they also pose nutritional challenges, particularly concerning calcium, iron, vitamin B12, zinc, and long-chain omega-3 fatty acids like EPA and DHA [1, 24]. These deficiencies can result in adverse health outcomes, including compromised bone health, anemia, impaired cognitive and immune function, and increased fracture risk. One of the most clinically significant outcomes is the risk of osteoporosis due to inadequate calcium and protein intake. Multiple studies have shown that vegetarians, especially women and older adults, may have lower bone mineral density and a higher incidence of fractures compared to omnivores [25]. Similarly, deficiencies in vitamin B12 and iron are common in plant-based diets and may lead to megaloblastic anemia and symptoms such as fatigue and neurological dysfunction, especially in vulnerable populations like children, pregnant women, and the elderly [24]. To address these clinical concerns, recent advancements include the fortification of plant-based foods with essential nutrients and the use of novel supplementation strategies. Algal-derived DHA and EPA supplements, fortified plant milks, cereals, and calcium-set tofu are among the innovations designed to bridge nutritional gaps in vegan and vegetarian populations [1, 42]. However, these strategies must be accompanied by regular clinical monitoring, including dietary assessments and the evaluation of serum biomarkers such as ferritin, vitamin B12, and 25-hydroxyvitamin D. Looking ahead, it is crucial for healthcare professionals to incorporate nutritional education and personalized dietary planning into routine care for individuals on plant-based diets. Public health policies should also promote awareness around the importance of nutrient-dense food choices and the need for appropriate supplementation when necessary. Further longitudinal studies are needed to assess the long-term health effects of fortified plant-based diets and to establish standardized clinical guidelines for nutritional management in this growing population group.

## Conclusions

In conclusion, while plant-based diets are often associated with numerous health benefits, including reduced risk of cardiovascular disease and certain cancers, they may also pose nutritional challenges if not properly managed. Individuals adhering to vegetarian or vegan diets are particularly susceptible to deficiencies in essential nutrients such as iron, zinc, calcium, vitamin B12, protein, and omega-3 fatty acids. These deficiencies can impair hemoglobin synthesis and bone remodeling processes, thereby increasing the risk of anemia and osteoporosis, especially in vulnerable groups such as pregnant women, children, and the elderly. Ensuring nutritional adequacy in plant-based diets requires deliberate dietary planning and, when necessary, the use of fortified foods or supplements. Moreover, attention must be given not only to nutrient intake but also to factors affecting nutrient bioavailability, such as the presence of dietary inhibitors (e.g., phytates and oxalates) and lifestyle habits. A well-balanced vegetarian or vegan diet, rich in whole foods and supported by evidence-based supplementation strategies, can support overall health and mitigate the risks associated with nutrient deficiencies. Public health initiatives and dietary education are thus essential to help individuals make informed decisions, enabling them to sustain plant-based eating patterns without compromising their long-term well-being.
